# Bone marrow aspirate concentrate for the treatment of osteochondral lesions of the talus: a systematic review of outcomes

**DOI:** 10.1186/s40634-016-0069-x

**Published:** 2016-11-04

**Authors:** Jorge Chahla, Mark E. Cinque, Jason M. Schon, Daniel J. Liechti, Lauren M. Matheny, Robert F. LaPrade, Thomas O. Clanton

**Affiliations:** 1 The Steadman Clinic, 181 West Meadow Drive, Suite 400, Vail, CO 81657 USA; 2 Steadman Philippon Research Institute, 181 West Meadow Drive, Suite 400, Vail, CO 81657 USA

**Keywords:** Bone marrow aspirate concentrate, Talar osteochondral lesion, BMAC treatment of osteochondral defects

## Abstract

**Background:**

The goal of this perform a systematic review on the outcomes of bone marrow aspirate concentrate (BMAC) for the treatment of chondral defects and osteoarthritis (OA) of the talus.

**Results:**

The systematic search performed identified 47 studies after duplicates were removed. After inclusion criteria were applied four studies were considered for insightful analysis for the treatment of focal chondral defects in the foot and ankle with the use of BMAC. Three studies were retrospective and one study was prospective in nature. One study was a comparative cohort study and three studies were case series.

**Conclusions:**

This review denotes that there exists an overwhelming paucity of long-term data and high-level evidence supporting BMAC for the treatment of chondral defects. Nonetheless, the evidence available showed varying degrees of beneficial results of BMAC for the treatment of ankle cartilage defects. The limited literature presented in this review demonstrates the need for more advanced, comparative studies to further investigate the efficacy, safety and techniques for BMAC in the treatment of OLTs. The authors recommend that BMAC therapy should be performed with careful consideration until the application and target population for this treatment are established.

## Background

Osteochondral lesions of the talus (OLTs) have become increasingly diagnosed and treated as advanced imaging technologies continue to improve the ability to detect cartilage defects (Potter et al. 2008; Verhagen et al. 2005; Leumann et al. 2011). In fact, some reports suggest that up to 50 % of acute ankle sprains and fractures may have associated OLTs (Savage-Elliott et al. 2014; Saxena and Eakin 2007). Common symptoms of OLTs include increased pain, stiffness, and functional limitations including decreases in activity level (Savage-Elliott et al. 2014).

Biological adjuncts such as bone marrow aspirate concentrate (BMAC) may be useful in increasing the longevity of cartilage repair procedures of the talus. Bone marrow aspirate consists of both mesenchymal stem cells (MSCs) and hematopoietic stem cells. Bone marrow aspirate concentrate has been theorized to facilitate regeneration of tissue, enhancing the quality of cartilage repair by increasing aggrecan content and tissue firmness (Sampson et al. 2013). As a result, BMAC promotes a potentially healthy environment for hyaline cartilage growth and repair, while minimizing the formation of fibrocartilage (Fortier et al. 2011; Smyth et al. 2012; Kennedy and Murawski 2011).

These concepts have now been studied in animal models, which have initially shown promising results and a limited complication profile in regards to BMAC (Fortier et al. 2010; Saw et al. 2009). The addition of BMAC to bone marrow stimulation (BMS) techniques significantly improved cartilage healing compared to BMS alone. Specifically, cartilage defects healed with a higher content of hyaline cartilage (collagen type II), more glycosaminoglycan, and better overall histological organization (Saw et al. 2009; Fortier et al. 2010). These results have led to the investigation of BMAC for the treatment of OLTs in human patients (Smyth et al. 2012). Moreover, BMAC is currently one of the few, United States Food and Drug Administration (FDA)-approved forms of delivering stem cells intraoperatively (McCright et al. 2009).

The overall evidence for treating chondral diseases using BMAC is limited and highly heterogeneous with respect to indications, timing, and results. However, outcomes following the use of BMAC for the treatment of OLTs have been previously documented (Hannon et al. 2016; Giannini et al. 2009; Kennedy and Murawski 2011); (Giannini et al. 2013). The purpose of this study was to systematically review the literature regarding indications, outcomes, complications and safety profile following the use of BMAC for the treatment of OLTs.

## Methods

### Article identification and selection

This study was conducted in accordance with the 2009 Preferred Reporting Items for Systematic Review and Meta-Analysis (PRISMA) statement (Moher et al. 2009). A systematic review of the literature regarding the existing evidence for outcomes for the treatment of chondral defects and osteoarthritis of the talus with BMAC was performed using the Cochrane Database of Systematic Reviews, the Cochrane Central Register of Controlled Trials, PubMed (1980–2016), and MEDLINE (1980–2016). The queries were performed in May 2016.

The literature search strategy included the following:

- Search Term 1: (“bone marrow”[MeSH Terms] OR “bone marrow”[All Fields]) AND (“aspirate”[All Fields] OR “concentrate”[All Fields]) AND (“ankle”[All Fields] OR “ankle”[MeSH Terms] OR “foot”[All Fields] OR “foot”[MeSH Terms])
- Search Term 2: bone[All fields] AND marrow[All fields] AND aspirate[All fields] AND (“ankle”[Mesh Terms] OR (“ankle”[All fields] AND “joint”[All fields]) OR “ankle joint” [All fields])

Inclusion criteria were as follows: BMAC for the treatment of cartilage defects of the ankle, English language, human studies with a follow-up greater than 12 months. Exclusion criteria consisted of cadaveric studies, animal studies, basic science articles, editorials articles, surveys, special topics, letters to the editor, personal correspondence, studies that did not include the talus or BMAC for treatment, of other pathologies not related to the cartilage.

Two investigators (initials blinded for review) independently reviewed the abstracts from all identified articles. Full-text articles were obtained for review if necessary to allow further assessment of inclusion and exclusion criteria. Additionally, all references from the included studies were reviewed and reconciled to verify that no relevant articles were missing from the systematic review.

### Data collection

The level of evidence of the studies was assigned according to the classification as specified by (Wright et al. 2003). The following information was extracted and recorded from the included studies: patient demographics, follow-up, and objective and subjective outcomes. For continuous variables (age, timing, follow-up, outcome scores), the mean and range were collected if reported. Data were recorded into a custom spreadsheet (Microsoft Corp) using a modified information extraction table (Harris et al. 2014).

#### Literature quality evaluation

A modified version of the Coleman methodology score (mCMS) to assess the quality of methodology in each study was utilized (Kon et al. 2009). The two part mCMS grades cartilage-related studies based on ten criteria; Part A: study size, mean follow-up, number of different surgical procedures, type of study, description of surgical procedure, postoperative rehabilitation, inclusion subjects’ MRI outcome and inclusion subjects’ histological outcome; Part B: outcome criteria, procedure for assessing clinical outcomes and description of subject selection process. The maximum score of the mCMS is 100, which indicates that a study largely avoids chance, biases and confounding factors. Two authors (initials blinded for review) independently reviewed and scored each study according to the proposed methodology.

## Results

### Study selection

**Fig. 1:**
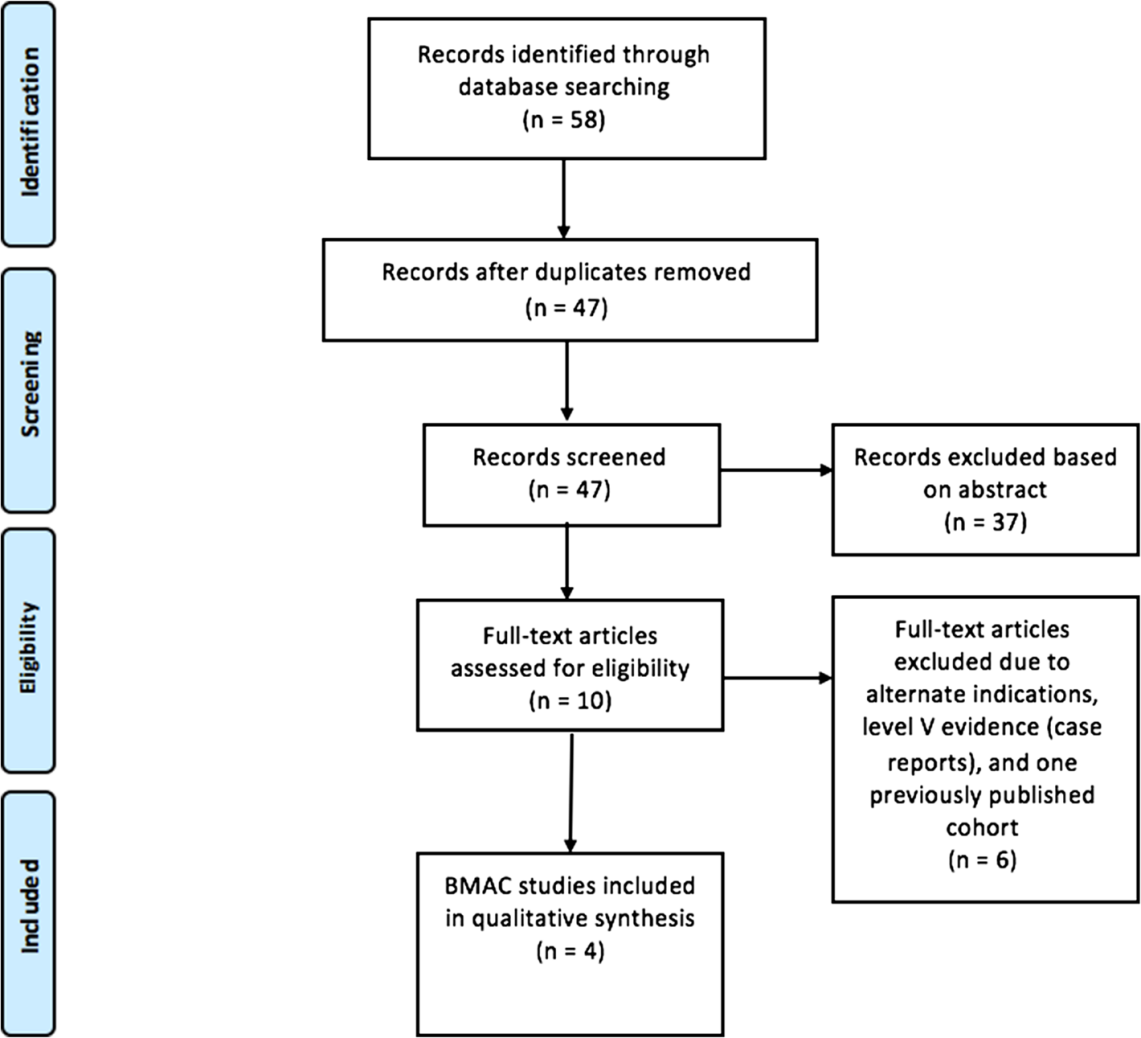
PRISMA flowchart of the included studies

The systematic search performed using the previously mentioned keywords identified 47 studies after duplicates were removed. Of these, 37 were basic science studies, cadaveric studies, or studies unrelated to our topic, leaving 10 articles. Of the remaining studies, 3 reported on alternate indications and use of BMAC such as fracture healing, 2 were case reports and 1 study published on the same patient cohort published in a previous study. After applying all exclusion criteria, 4 studies for the treatment of focal chondral defects in the ankle with the use of BMAC were considered. Three studies were retrospective (Giannini et al. 2013; Giannini et al. 2009; Kennedy and Murawski 2011) and one study (Hannon et al. 2016) was prospective in nature. One study was a comparative cohort study (Hannon et al. 2016) and three studies were case series (Giannini et al. 2013; Giannini et al. 2009; Kennedy and Murawski 2011). There was considerable heterogeneity of indications, subjective outcomes measures, and objective data (e.g. MRI, second look arthroscopy) among the included studies. Figure 1 demonstrates a PRISMA flowchart of the selection criteria of the studies found with our search.

### Demographics

The review included a total of 184 patients with a mean age of 29.5 years in the included studies. Average follow-up of the included studies was 34.3 months (range, 24 to 77 months). Lesion location was also well distributed among studies, with a higher incidence of medial talar dome lesions (*n* = 146) compared to lateral talar dome lesions (*n* = 56). Two studies (Hannon et al. 2016; Kennedy and Murawski 2011) further specified lesion location with the 9-zone anatomic localization scheme on MRI proposed by (Elias et al. 2007), which can be found in Table 1.

**Table 1:** Patient demographics

Article	Study Size	Mean Age	Mean Follow-up (months)	Location and Size	Previous Treatment	Marrow harvesting and preparation	Focal cartilage defect severity
Hannon et al. 2016	*n* = 34 12 BMS 22 BMAC + BMS	BMS: 39 (18–60) BMAC + BMS: 35 (12–68)	BMS: 77.3 (46–100) BMAC + BMS: 48.3 (34–82)	Mean area: BMS: 111.2 mm^2^ CL (3), PL (2), AM (1), CM (1), PM (5) BMAC + BMS: 103 mm^2^ AL (3), CL (7), AM (2), CM (8), PM (2)	None reported	Approx. 60 mL of bone marrow aspirate harvested from ipsilateral iliac crest and concentrated using an Arteriocyte Magellan Autologous Platelet Separator System to obtain approximately 3 mL of BMAC.	Mean talar osteochondral lesion area: BMS:111.2 mm^2^; BMAC/BMS: 103 mm^2^
Kennedy and Murawski 2011	*n* = 72	34.19 (16–85)	28.02 (12–64)	Mean AP size: 11.2 mm (6–20 mm) Mean medial-lateral size: 10.74 mm (7–20 mm) 49 medial talar dome (2 AM, 33CM, 14 PM), 23 Lateral talar dome (6 AL, 9 CM, 8 PL)	None reported	Approx. 60 mL of bone marrow aspirate harvested from anterior ilium of the ipsilateral iliac crest and concentrated using a commercial BMAC centrifuge system to obtain roughly 4 mL of BMAC.	Mean anterior-posterior size: 11.2 mm (6–20 mm), mean medial-lateral size 10.74 mm (7–20 mm) >6 mm diameter talar osteochondral lesion
Giannini et al. 2009	*n* = 48	28.5 +/− 9.5	29 (24–35)	Mean size: 2.07 +/− 0.48 cm^2^, Mean depth 4.0 +/− 0.9 mm; 39 Medial talar dome 9 Lateral talar dome	8 microfracture 5 debridement 2 autologous chondrocyte implantation	60 mL of bone marrow harvested from the posterior iliac crest and concentrated with a Harvest Tech Smart PReP using the Harvest BMAC kit to obtain 6 mL of BMC.	Chronic type II (>1.5 cm^2^, <5 mm deep), mean size 2.07 +/− 0.48 cm^2^, mean depth 4.0 +/− 0.9 mm
Giannini et al. 2013	*N* = 20	Not Reported	36	MFC (14), LFC (4),	None Reported	60 mL of bone marrow harvested from the posterior iliac crest and concentrated with a Harvest Tech Smart PReP using the Harvest BMAC kit to obtain 6 mL of BMC.	Post-traumatic grade III. IV osteochondral lesions

Abbreviations are as follows: *BMAC* bone marrow aspirate concentrate, *BMS* bone marrow stimulation, *AL* anterolateral, *CL* centrolateral, *PL* posterolateral, *AM* anteromedial, *CM* centromedial, *PM* posteromedial

### Indications

All included studies utilized BMAC injection as an adjunct for treatment of OLTs. However, procedures between studies were variable with one study performing only microfracture both with and without BMAC augmentation,(Hannon et al. 2016) one study performing only OAT with BMAC,(Kennedy and Murawski 2011) and two studies performing arthroscopic debridement with BMAC placement with one of two scaffolds (Giannini et al. 2013; Giannini et al. 2009). The study, in which OAT was performed, lesion size was at least 6 mm in diameter (Kennedy and Murawski 2011). In the studies by (Giannini et al. 2009; Giannini et al. 2013) scaffolds were used for large, chronic Type II lesions (>1.5 cm^2^ area, < 5 mm deep).

### BMAC extraction and processing

The quantity of bone marrow aspirate extracted was consistent in all studies (60 mL) from the anterior iliac crest in two studies (Hannon et al. 2016; Kennedy and Murawski 2011) and from the posterior iliac crest in the remaining two studies (Giannini et al. 2013; Giannini et al. 2009). Processing systems utilized were heterogeneous: (Hannon et al. 2016) utilized an Arteriocyte Magellan Autologous Platelet Separator System (obtaining 3 ml of BMAC). Giannini (Giannini et al. 2009; Giannini et al. 2013) utilized the Harvest Tech Smart PReP to obtain 6 mL of BMAC. Finally, the centrifuge used by Kennedy (2011) was not reported in their study although they obtained 4 mL as a result of the BMA processing (Table 2).

**Table 2:** Outcome study reported data metrics

Study	Study Size	Treatment	Additional Treatment	Preoperative AOFAS	Postoperative AOFAS	Preoperative FAOS	Postoperative FAOS	Preoperative SF-12	Postoperative SF-12	Radiologic findings	Second-look arthroscopy	Complications
Hannon et al. 2016	*N* = 34	BMS alone Vs BMS with BMAC	None			BMS 54.8 BMS + BMAC 60.6	BMS 68.3 BMS + BMAC 77.6	BMS 38.5 BMS + BMAC 42.5	BMS 55.3 BMS + BMAC 61.9	Total MOCART score BMS: 55.8 BMAC + BMS: 73.0 (BMAC with significantly greater defect filling, border repair integration, and surface tissue repair at 2 year follow-up)	Not performed	BMS: 1 subchondral cyst formation BMAC/BMS: 2 superficial peroneal nerve dysesthesias
Kennedy and Murawski 2011	*N* = 72	Osteochondral autograft soaked in BMAC with synthetic filler soaked in BMAC	None			52.67	86.2	59.4	88.6	MRI: In 1 ankle, small cyst formation beneath graft site at 28 months	Not performed	3 donor site knee pain, 1 cyst growth beneath graft site
Giannini et al. 2009	*N* = 48	Collagen scaffold + BMA OR Hyaluronic acid membrane scaffold + BMA	17 osteophytectomy, 2 synovectomy, 2 loose body extraction, 1 calcaneal osteotomy	64.4 ± 14.5	6 months 83.3 ± 8.7 12 months 88.9 ± 8.2 18 months 89.7 ± 8.5 24 months 91.4 ± 7.7					2 patients at 12 months showed hypertrophy of new tissue on MRI; at 24 months all patients showed restored focal cartilage layer at defect site on MRI	5 patients evaluated at mean 13 months (12–14); 3 asymptomatic patients with newly formed cartilage 2 symptomatic patients with hypertrophy of new tissue; All patients with smooth, complete and healthy cartilage integration	1 superficial infection at portal
Giannini et al. 2013	*N* = 20	Collagen scaffold + BMA OR Hyaluronic acid membrane scaffold + BMA	None	63.73 ± 14.13	48 (±6) months 82.19 ± 17.04					20 patients under went MRI T2 Mapping: 45 % Complete defect filling 45 % Incomplete >50 % filling 10 % Incomplete <50 % 78 % had hyaline like cartilage at latest follow-up	Not performed	None Reported

### Patient reported outcomes

#### Post-procedure imaging, second-look arthroscopy, and quality of the repair tissue

Two studies performed postoperative MRIs at a minimum 24 months follow-up to assess the quality of the repair (Hannon et al. 2016; Giannini et al. 2009); (Hannon et al. 2016) utilized the magnetic resonance observation of cartilage repair tissue (MOCART) (Marlovits et al. 2006) score and found significantly higher scores in the BMS with BMAC group compared to BMS alone. Specifically, they reported significantly improved defect filling, border repair integration and surface tissue repair along with far less evidence of fissuring and fibrillation in OLTs treated with BMAC (Hannon et al. 2016). At 2 year follow-up, (Giannini et al. 2009) reported that all patients showed evidence of restored cartilage layer at the OLTs defect site on MRI. Additionally, (Giannini et al. 2009) performed second-look arthroscopy in 5 patients at a mean 13 months. Three of these patients were asymptomatic and the other 2 patients reported symptoms of continued pain. Second-look arthroscopy showed evidence of chondral hypertrophy in the 2 symptomatic patients, but all patients showed evidence of complete and healthy cartilage integration. Histological and immunohistochemical analysis of three patient biopsy samples collected at 12 month revealed various degrees of hyaline cartilage reformation with visible chondrogenic growth, increased hyaline cartilage and proteoglycan content (Giannini et al. 2009). Giannini et al. also utilized MRI T2/MOCAT score in 20 patients from their 49 patient four year follow up cohort. They found no significant relationship between MOCART score parameters and patient outcomes at 48 months (Giannini et al. 2013).

#### Return to activity

Giannini (2009) reported that 94 % of patients returned to low impact sports activity at a mean 4.4 months and 77 % of patients returned to high impact sports activity at a mean 11.3 months. The same authors reported that 73 % of the 36 patients playing sports before surgery were able to return to sports in a different study (Giannini et al. 2013). They also reported that 22 % of these 36 patients were able to return to sport, but at a lower level than before surgery (Giannini et al. 2013) Kennedy et al.(2011) reported that 95 % of patients who had undergone OAT with BMAC augmentation returned to their pre-symptom level of sporting activity at a mean 13 weeks.

#### Safety

There were 8 complications reported in the included studies. The most common complication was donor site knee pain in 3 patients in which the lateral femoral condyle was used as the graft site (Kennedy and Murawski 2011). There were two reports of subchondral cyst formation, one at the graft harvest site (Kennedy and Murawski 2011) and one in the OLTs lesion (Hannon et al. 2016) In patients who received BMAC in addition to BMS, 2 superficial peroneal nerve dysesthesias were reported (Hannon et al. 2016). Lastly, there was 1 patient who developed a superficial infection at one of the arthroscopic portal sites (Giannini et al. 2013).

#### Literature methodological quality assessment

The mean score of the included studies was 57 out of 100 points using the Kon-Verdonk modified Coleman methodology score (Kon et al. 2009). The mean score was 64 points for the Hannon et al. (2016) study, 62 points for both of the Giannini (2013; Giannini et al. 2009) studies and 40 points for the Kennedy et al.(2011) study.

## Discussion

The most important finding of this review was that there was a scarcity of information in the literature on the use of BMAC for the treatment of OLTs with highly heterogeneous indications, associated procedures and outcome measurements. However, the reviewed studies showed varying degrees of beneficial outcomes for the treatment of moderately sized chondral defects with no major complications reported. While the three studies included in this review were of moderate to high quality, as determined by the mCMS, only one study was prospective and used a control group (Hannon et al. 2016). Despite increased use, development, and popularity of BMAC, additional comparative studies are certainly necessary to provide additional support for its efficacy in the treatment of OLTs.

Favorable short- and medium-term outcomes for the treatment of focal cartilage defects and osteoarthritis have been reported in other joints, such as the knee (Bhatia et al. 2015). However, the indications, delivery method, and composition of BMAC have been heterogeneous. In the present studies (Giannini et al. 2009; Kennedy and Murawski 2011; Hannon et al. 2016; Giannini et al. 2013). BMAC has been used in conjunction with either synthetic scaffolds, OAT or BMS and resulted in good short-term clinical outcomes. BMAC has also been delivered with a porcine collagen matrix and hyaluronic acid membranes (Buda et al. 2013). Furthermore, three studies (Giannini et al. 2009; Kennedy and Murawski 2011; Giannini et al. 2013) reported that the vast majority of patients were able to successfully return to athletic activity. Certainly, further studies are warranted to determine the optimal delivery and composition of BMAC.

Follow-up MRI in three of the included studies (Giannini et al. 2009; Hannon et al. 2016; Giannini et al. 2013) supported good cartilage defect filling. Hannon et al. (Hannon et al. 2016) reported significantly greater MRI findings among patients treated with BMAC and BMS compared to patients treated with BMS alone. The findings reported by Hannon et al. are similar to those reported by Fortier et al.(2010) In a comparative study of microfracture with or without BMAC augmentation for the treatment of full thickness cartilage defects in an equine knee model, Fortier et al.(2010) reported improved defect filling, integration of repair tissue, collagen orientation and increased glycosaminoglycan and type II collagen content in the BMAC group. Only one study (Giannini et al. 2009) in this review performed second-look arthroscopy. All 5 patients showed evidence of complete and healthy cartilage integration at a mean 13 months; however, evidence of hypertrophic chondral growth in 2 symptomatic patients raises particular concern.

In the reviewed studies, few complications were reported, and no complications were attributed to the BMAC injection itself. Only superficial infection at a portal site were reported in the study utilizing scaffold supported BMAC (Giannini et al. 2009). Following BMAC soaked OAT, three patients suffered from donor site-morbidity related pain and one MRI at 28 months follow-up revealed cyst formation beneath the graft site (Kennedy and Murawski 2011). Two patients complained of superficial peroneal nerve dysesthesia after microfracture and BMAC treatment (Hannon et al. 2016). There were no reports of neoplasia or excessive bone formation. However, Giannini et al.(2009) did report two symptomatic patients with evidence chondral hypertrophy at second-look arthroscopy. Overall, bone marrow aspirate appears to be a safe biologic adjunct.

Clanton et al.(2014) reported short-term results of 7 patients (mean age: 43.7 years) at a mean follow-up of 8.4 months (range: 6.3 to 12.6) following arthroscopic treatment of OLTs with microfracture and a mixture of cartilage extracellular matrix augmented with BMAC. Mean Foot and Ankle Disability Index (FADI) Activities and Daily Living (Mesfar and Shirazi-Adl 2008) subscale scores improved from 64 (range: 39–89) preoperatively to 83 (range: 62–100) at follow-up. Mean FADI sports subscale improved from 29 (range: 0–47) to 53 (range: 22–100) postoperatively. Mean FADI total score improved from 56 (range: 33–79) to 76 (range: 52–100) at follow-up. This study was excluded from our systematic review due to the follow-up time of less than 12 months. However, these preliminary outcomes are important for establishing BMAC as a viable technique for OLT treatment.

The authors recognize some limitations of this systematic review. In the evaluated studies, BMAC was used to augment a variety of surgical techniques for treatment of OLTs. Therefore, the results of this study do not demonstrate the efficacy and safety of BMAC as an isolated therapy. The heterogeneity in the presentation of results diminishes the ability to compare subjective and objective outcomes across studies. Similarly, there was limited consistency in the quantitative description of osteochondral lesion size. The lack of control groups in the presented studies decreases the ability to evaluate relative efficacy of the procedures. Furthermore, the relatively short-term results of the included studies are not representative of the long-term implications of BMAC treatment for OLTs and this should be considered when interpreting this review. Lastly, inherent to any systematic review, there is the possibility that not all relevant articles were identified through the used search terms and literature review.

## Conclusion

This review denotes that there exists an overwhelming paucity of long-term data and high-level evidence supporting this treatment method. Nonetheless, the evidence available showed varying degrees of beneficial results of BMAC for the treatment of ankle cartilage defects. The limited literature presented in this review demonstrates the need for more advanced, comparative studies to further investigate the efficacy, safety and techniques for BMAC in the treatment of OLTs. The authors recommend that BMAC therapy should be performed with careful consideration until the application and target population for this treatment are established.

## Abbreviations

BMAC: Bone marrow aspirate concentrate
BMS: Bone marrow stimulation
FDA: Food and drug administration
mCMS: Modified version of the Coleman methodology score
MOCAT: Magnetic resonance observation of cartilage repair tissue
MRI: Magnetic resonance imaging
MSC: Mesenchymal stem cells
OLT: Osteochondral lesions of the talus
PRISMA: Preferred reporting items for systematic review and meta-analysis
