# Erratum to: Bone marrow aspirate concentrate for the treatment of osteochondral lesions of the talus: a systematic review of outcomes

**DOI:** 10.1186/s40634-016-0074-0

**Published:** 2016-12-23

**Authors:** Jorge Chahla, Mark E. Cinque, Jason M. Schon, Daniel J. Liechti, Lauren M. Matheny, Robert F. LaPrade, Thomas O. Clanton

**Affiliations:** 1 The Steadman Clinic, 181 West Meadow Drive, Suite 400, Vail, CO 81657 USA; 2 Steadman Philippon Research Institute, 181 West Meadow Drive, Suite 400, Vail, CO 81657 USA

Unfortunately, after publication of this article (Chahla et al. 2016), it was noticed that the name of the third author was incorrectly spelled “Jason M. Shon”. The correct spelling is Jason M. Schon; the corrected author list can be seen above and the original article has been updated to reflect this change.
